# Hyperspectral Characteristics and SPAD Estimation of Wheat Leaves under CO_2_ Microleakage Stress

**DOI:** 10.3390/s24154776

**Published:** 2024-07-23

**Authors:** Liuya Zhang, Debao Yuan, Yuqing Fan, Renxu Yang

**Affiliations:** College of Geoscience and Surveying Engineering, China University of Mining and Technology (Beijing), Beijing 100083, China; sqt2200204094@student.cumtb.edu.cn (L.Z.); zqt2200204120@student.cumtb.edu.cn (Y.F.); sqt2200204091@student.cumtb.edu.cn (R.Y.)

**Keywords:** microleakage, SPAD, wheat, fractional order differentiation, multiple linear regression

## Abstract

To non-destructively and rapidly monitor the chlorophyll content of winter wheat leaves under CO_2_ microleakage stress, and to establish the quantitative relationship between chlorophyll content and sensitive bands in the winter wheat growing season from 2023 to 2024, the leakage rate was set to 1 L/min, 3 L/min, 5 L/min, and 0 L/min through field experiments. The dimensional reduction was realized, fractional differential processing of a wheat canopy spectrum was carried out, a multiple linear regression (MLR) and partial least squares regression (PLSR) estimation model was constructed using a SPA selection band, and the model’s accuracy was evaluated. The optimal model for hyperspectral estimation of wheat SPAD under CO_2_ microleakage stress was screened. The results show that the spectral curves of winter wheat leaves under CO_2_ microleakage stress showed a “red shift” of the green peak and a “blue shift” of the red edge. Compared with 1 L/min and 3 L/min, wheat leaves were more affected by CO_2_ at 5 L/min. Evaluation of the accuracy of the MLR and PLSR models shows that the MLR model is better, where the MLR estimation model based on 1.1, 1.8, 0.4, and 1.7 differential SPAD is the best for leakage rates of 1 L/min, 3 L/min, 5 L/min, and 0 L/min, with validation set R^2^ of 0.832, 0.760, 0.928, and 0.773, which are 11.528, 14.2, 17.048, and 37.3% higher than the raw spectra, respectively. This method can be used to estimate the chlorophyll content of winter wheat leaves under CO_2_ trace-leakage stress and to dynamically monitor CO_2_ trace-leakage stress in crops.

## 1. Introduction

With the acceleration of industrialization and increasing energy demand, CO_2_ emissions have become a global concern [1,2,3]. CO_2_ geological storage has become an indispensable technological pathway to achieve the goal of a dual-carbon strategy because it can control CO_2_ emissions while considering socioeconomic development [4]. However, in the geological storage of CO_2_, there is a risk of leakage or seepage of the stored CO_2_ outside the storage area, which may have serious impacts on the environment, as well as the surrounding organisms [5]. In agricultural production, CO_2_ microleakage occurs because of facility-based agriculture and improper disposal of industrial waste, and its potential stress on crop growth may occur [6].

Wheat is one of the most widely grown food crops worldwide [7,8], but its growth and yield are influenced by a variety of environmental factors. In recent years, it has been shown that changes in CO_2_ concentration can significantly affect the physiological characteristics of wheat, including photosynthetic efficiency and chlorophyll content [9]. Among them, chlorophyll content (SPAD), an important indicator of plant photosynthetic capacity and growth, is crucial for the yield and quality of wheat [10]. Therefore, it is of great theoretical and practical significance to study changes in the chlorophyll content of wheat under CO_2_ microleakage stress and its monitoring methods.

With the rapid development of remote sensing technology, the quantitative monitoring of CO_2_ stress over large areas will become possible. Remote sensing technology plays a significant role in monitoring crop growth and development in large-scale farmlands [11]. Hyperspectral remote sensing technology has the advantages of high spectral resolution, strong band continuity, and broad information, which can obtain data on fine changes in the physiochemistry of crop leaves [12], and the monitoring has the characteristics of being non-destructive, continuous, and fast. Rapid, non-destructive, and accurate quantitative monitoring of CO_2_ microleakage using hyperspectral technology is one of the most important methods for CO_2_ stress diagnosis. Currently, remote sensing spectral monitoring of plant physiological parameters has been extensively employed in the domains of biotic and abiotic stresses. Xu et al. [13] investigated the hyperspectral traits of cotton leaves under waterlogging stress and the estimation model of chlorophyll content, and acquired the estimation model of chlorophyll content of cotton leaves under waterlogging stress. Xia et al. [14] probed into the spectral sensitive interval of corn leaves under heavy metal copper stress and proposed a novel vegetation index based on sensitive bands for heavy metal copper stress, which is conducive to the future monitoring research of heavy metal copper in corn leaves. Goswami et al. [15] carried out hyperspectral research on rice under N, CO_2_, and temperature stresses and discovered that the red edge position in the spectral characteristic parameters is an excellent indicator for crop stress monitoring. When plants are under stress, the red edge position shifts towards the short-wave direction in an extremely sensitive manner, which is also in line with the current spectral research findings of plant stress. Noomen and Skidmore’s [16] research specifically points out that CO_2_ exposure significantly alters the distance between the red edge position (REP) and the yellow edge position (YEP) of plant leaves, which is a direct manifestation of the impaired photosynthetic capacity of plants. The research by Lakkaraju et al. [17] further confirmed the efficacy of hyperspectral remote sensing in monitoring and evaluating the impact of environmental stresses on plants by analyzing the relationship between various vegetation indices and soil CO_2_ concentration. These vegetation indices, such as the Structure Insensitive Pigment Index (SIPI) and Chlorophyll Normalized Difference Vegetation Index (Chl NDI), all exhibit high sensitivity in identifying stresses related to elevated CO_2_. To conclude, although remote sensing spectral technology has explored numerous biotic and abiotic stresses, spectral research on CO_2_ stress is relatively infrequent, particularly for spectral research on CO_2_ stress of crops.

The estimation of crop chlorophyll content is a hot research topic at home and abroad, mostly based on the correlation between original spectra [18,19], spectral index construction [20,21,22] and integer-order spectral transformation [19], and chlorophyll content, and it is difficult to dig the information implied by the hyperspectral data by relying only on these parameters. Fractional order discretization can improve the utilization rate of spectral information and enhance the sensitivity of spectral information to crop physicochemical parameters [23,24]. Li et al. [25] carried out fractional order discretization of unmanned aerial vehicle (UAV) hyperspectral data, which successfully discretized the local information, effectively reduced the influence of environmental background factors, improved the correlation with the chlorophyll content of potato of different fertility stages, and accurately estimated the chlorophyll content of potato of different fertility stages based on machine learning. Liu et al. [26] found that the optimal fractional order differential spectra corresponding to potato biomass at different fertility periods appeared at different orders, and the correlation was significantly improved compared with the integer order, which greatly improved the estimation accuracy of potato above-ground biomass. Comparison of previous studies revealed that, on the one hand, there have been more studies on the construction of chlorophyll content estimation models using hyperspectral technology, but fewer studies on the hyperspectral modelling of chlorophyll content estimation under CO_2_ microleakage stress; on the other hand, the screening of representative spectral bands is the basis for the construction of a hyperspectral estimation model, in which SPA has been proven by more researchers to be able to efficiently extract the characteristic spectral bands and improve the accuracy of the estimation model [27,28].

In this study, we obtained winter wheat canopy hyperspectral reflectance data and SPAD under four CO_2_ leakage rate levels in winter wheat through field experiments. First, we processed the raw spectra using fractional-order differential processing, introduced the SPA to select the sensitive feature bands, and then used the MLR and PLSR models to make a hyperspectral estimation of SPAD of wheat under microleakage stress to assist in the determination of CO_2_ microleakage information.

## 2. Materials and Methods

### 2.1. Overview of the Research Area

**Figure 1:**
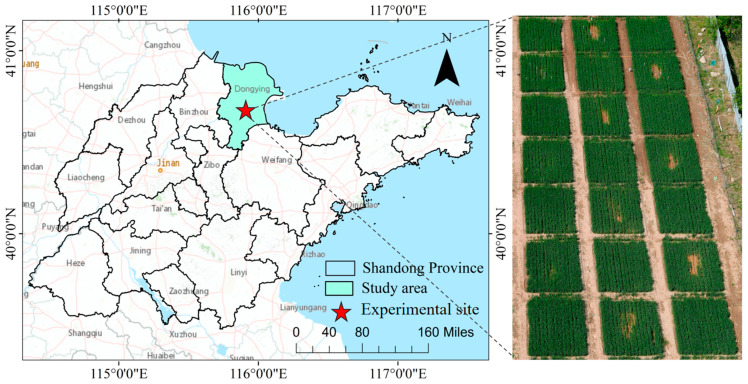
Research zone.

**Figure 2:**
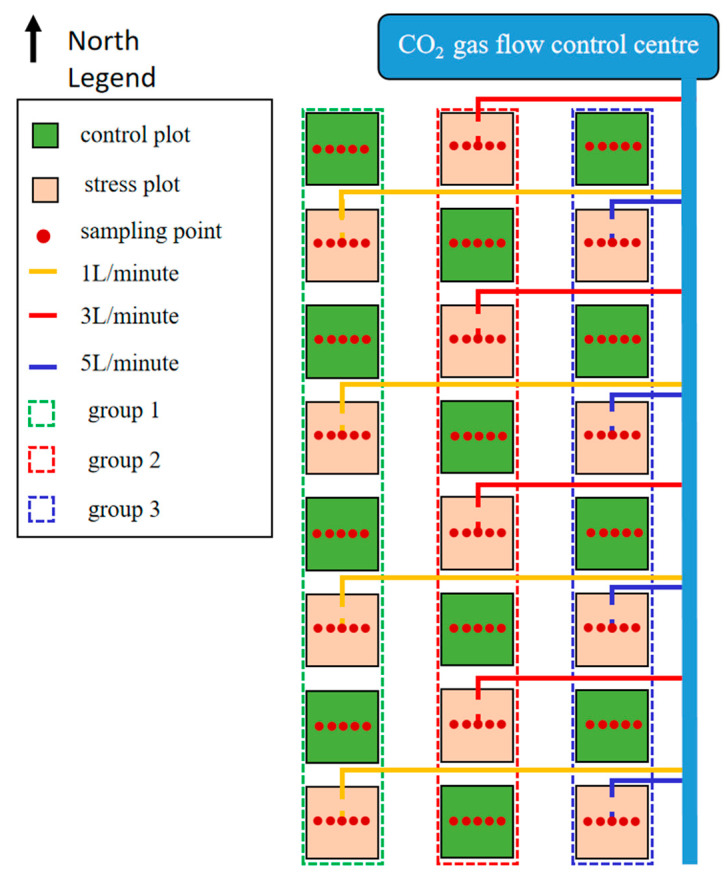
Spatial distribution of experimental sites.

**Figure 3:**
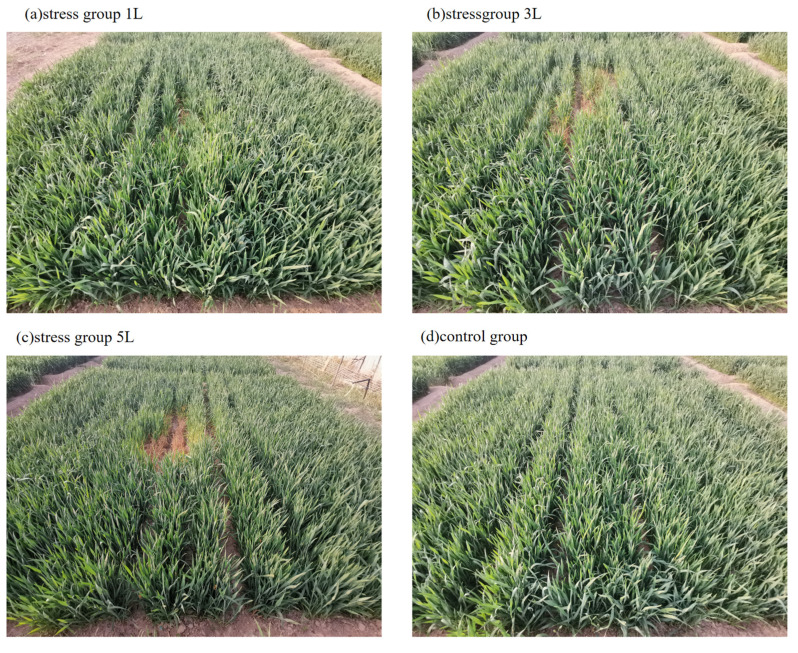
Different leakage stress rate trials in wheat. (**a**) stress group 1 L, (**b**) stress group 3 L, (**c**) stress group 5 L, (**d**) control group.

The study area is located in Dong ying District, Dong ying City, Shandong Province (118°7′ E, 32°2′ N), as shown in Figure 1. The basic climatic characteristics of the study area are cold winters and hot summers, four distinct seasons with a multi-year average temperature of 12.8 °C, and an average annual precipitation of 555.9 mm, which is mostly concentrated in the summer months and accounts for 65% of the annual precipitation, with large inter-annual variations in precipitation. The experiment was carried out in 2023, with winter wheat sown in November and harvested in June of the following year, and the experimental area was divided into 30 field plots, each with an area of 4 m × 4 m. The experiment was set up with four leakage rates as shown in Figure 2 and Figure 3, which were 0 L/min (control group), 1 L/min, 3 L/min, and 5 L/min, respectively. Other management measures followed local winter wheat field management standards.

### 2.2. Experimental Field Design

In the experimental plot, in order to safely and effectively transport CO_2_, corrosion-resistant copper pipes were adopted as the gas transmission medium in the initial stage. As the pipeline approached the plot area, in order to adapt to the plot environment and reduce costs, lightweight and durable PVC pipes were used instead. The PVC pipe starts from the northern edge of the plot, with an inclined posture forming a 30° with the vertical line, and penetrates about 60 cm deep into the centre of the plot to achieve precise gas distribution. At the end of the PVC pipe, eight evenly distributed small holes are carefully designed, and these holes face in different directions, aiming to ensure that CO_2_ can form a uniform concentration distribution when leaked and avoid local accumulation. At the same time, to prevent soil particles from entering and blocking these small holes, a layer of fine gauze is specially covered at each small hole, which plays a good protective role. To precisely control the leakage rate of CO_2_, a programmable logic controller (PLC) was introduced as the core for remote monitoring and adjustment. Through this advanced system, the leakage speed of CO_2_ was precisely set at 1 L/min, 3 L/min, and 5 L/min, and all-weather uninterrupted automatic control was achieved. Since the start of the experiment, this stable and continuous leakage process continued until the experiment was successfully completed.

In the experimental design, in order to ensure the accuracy of the experimental results, both the control group and the stress group followed a strict and identical management process, covering key links such as watering, weeding, fertilization, and pest control, aiming to provide a unified environment conducive to plant growth. The only significant difference between the two is that the experimental group plot introduced the specific variable of CO_2_ injection, while the control group did not carry out this operation in order to explore the specific impact of CO_2_ on plant growth.

### 2.3. Data Acquisition

**Figure 4:**
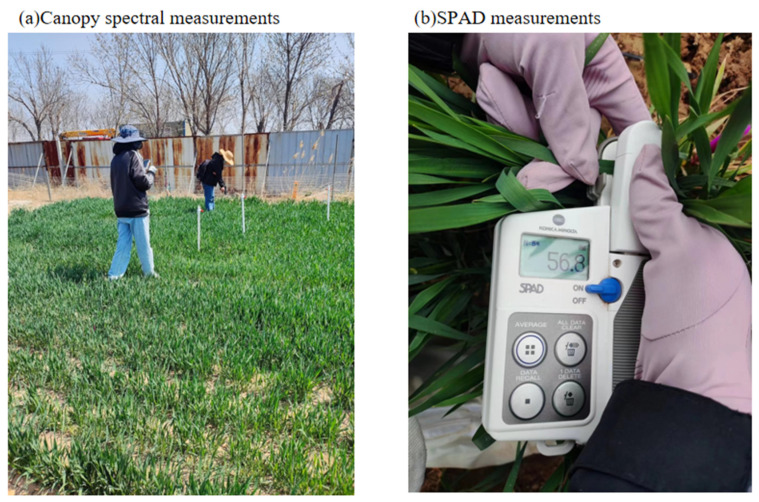
Data Measurements. (**a**) Canopy spectral measurements using the SVC HR-1024i spectrometer. (**b**) SPAD measurements with the SPAD-502 handheld chlorophyll meter.

As shown in Figure 4a, canopy spectra were measured using an SVC HR-1024i spectrometer (Spectra Vista Corporation, Poughkeepsie, NK, USA) [29] to detect canopy spectral reflectance in the wavelength range of 350–2500 nm with a fibre optic field of view of 25°. Five sampling points were deployed in each experimental area, and the spectral reflectance measurements were performed on clear, windless, and cloudless days between 10:00 and 14:00 to minimize errors resulting from variations in illumination conditions [30]. Spectral data have different resolutions depending on the range of bands, specifically ≤3.3 nm in the interval 300–1000 nm, ≤9.5 nm in the interval 1000–1890 nm, and ≤6.5 nm in the interval 1890–2500 nm. In order to unify the analytical standards, the spectral resolution was resampled to 1 nm using the SVC professional software 7.1, and the optical fibre was accurately aligned with the canopy during the measurement process, maintaining a distance of about 1 m. The distance was maintained at approximately 1 m, with a single scan lasting approximately 3 s. Data were collected at five different sampling points for each plot to ensure that the results were comprehensive and representative. To eliminate the effect of noise in the spectral data, the study selected hyperspectral reflectance data in the range of 350–1350 nm for analysis and modelling. Winter wheat leaf chlorophyll was obtained using a SPAD-502 handheld chlorophyll meter (Minolta Corporation, Tokyo, Japan), and the mid-leaf part of each leaf sample was measured separately during the measurement process as shown in Figure 4b, and the average instrumental measurement from SPAD was used as the chlorophyll content of leaf samples. Canopy spectral reflectance was collected by randomly selecting 5 leaf samples at the canopy top of each field and taking the average value as the measured value of each sample point.

### 2.4. Methods of Data Processing

#### 2.4.1. Data Preprocessing

When measuring spectral data in the field, due to the influence of uncontrollable factors (such as light, wind, observation angle, etc.), spectral data may produce distortion or error, which affects the accurate reflection of actual feature and subsequent data analysis and modelling. Therefore, strict quality control of data measured using spectrometers such as the SVC HR-1024i is essential. This includes the removal of invalid bands, the exclusion of invalid samples, and the exclusion of pathological samples (samples with missing bands or obviously abnormal jumps). These quality control measures ensure the representativeness and accuracy of the spectral data, and provide a reliable basis for subsequent analyses.

Spectral data often contain “burr” noise due to the differences in energy responses, which reduce the signal-to-noise ratio and affect the analysis. In order to obtain a smooth spectral curve, it is necessary to carry out smoothing to eliminate noise and retain useful information. In this paper, the five-point weighted moving average smoothing method is adopted to achieve spectral smoothing while retaining the details through differentiated weight allocation.

#### 2.4.2. Fractional Order Differentiation

Fractional order differentiation is a fundamental mathematical operation with a wide range of applications in fields such as image enhancement processing and signal analysis [31]. Fractional order differentiation extends the concept of integer order differentiation, which is an area dedicated to the study of the mathematical properties and applications of arbitrary order differentiation [32,33]. The traditional integer order differentiation ignores some information related to the chlorophyll content, which affects the model accuracy. The fractional order differentiation can refine the local information of the hyperspectral data, and it can also effectively denoise and obtain the fine detail information. The mathematical order differentiation operation is mainly based on Grunwald–Letnikov (G-L) fractional order differentiation one-dimensional function difference expression to achieve, in order to mine the hyperspectral finer gradient information as well as to reduce the noise and interference that inevitably arises in the sampling process, the wheat leaf hyperspectral curves under different treatments, which are transformed with a fractional order differentiation between 0 and 2 with a step size of 0.1. A total of 21 fractional order differential transforms were obtained, and specific algorithmic procedures for the calculation of fractional order differentiation were implemented using Maltalb2019b.

#### 2.4.3. Successive Projections Algorithm

The successive projections algorithm (SPA) is programmed to screen out the feature bands from the sensitive region. SPA is an emerging dimensionality reduction method, which achieves substantial spectral dimensionality reduction and at the same time ensures that the covariance between the feature bands is minimized, and in recent years, it has been widely used in the research of crop hyperspectral information [34].

### 2.5. Methods of Modelling

In this study, multivariate linear regression (MLR) and Partial Least Squares Regression (PLSR) were used as a modelling method to construct the estimation model and validate it. MLR is an important method in multivariate statistical analysis, which is widely used due to its wide range of applications, ease of operation, and other characteristics [35]. PLSR is a linear regression method that combines multiple linear regression, typical correlation analysis, and principal component analysis, which can effectively overcome the difficulties that cannot be solved by general least squares regression analysis methods, and has obvious advantages for continuous spectral analysis, and is usually used to construct predictive models. The stability and predictive ability of the model is mainly evaluated using the highest Coefficient of determination (*R*^2^), Root mean square error (*RMSE*). The calculation formula is as follows:

(1) $$R^{2}=\frac{\sum_{i=1}^{n}{\left(\hat{y_{i}}-y_{i}\right)}^{2}}{\sum_{i=1}^{n}{\left(y_{i}-\bar{y}\right)}^{2}}$$

(2) $$RMSE=\sqrt{\frac{1}{n}\sum_{i=1}^{n}{\left(\hat{y_{i}}-y_{i}\right)}^{2\;}}$$

In the formula, $\hat{y_{i}}$ is the predicted value; $y_{i}$ is the observed value; $\bar{y}$ is the mean of the sample observations; n is the total number of samples; and i is the sample number.

## 3. Results

### 3.1. Effect of Different Concentrations of CO_2_ on SPAD in Wheat

**Figure 5:**
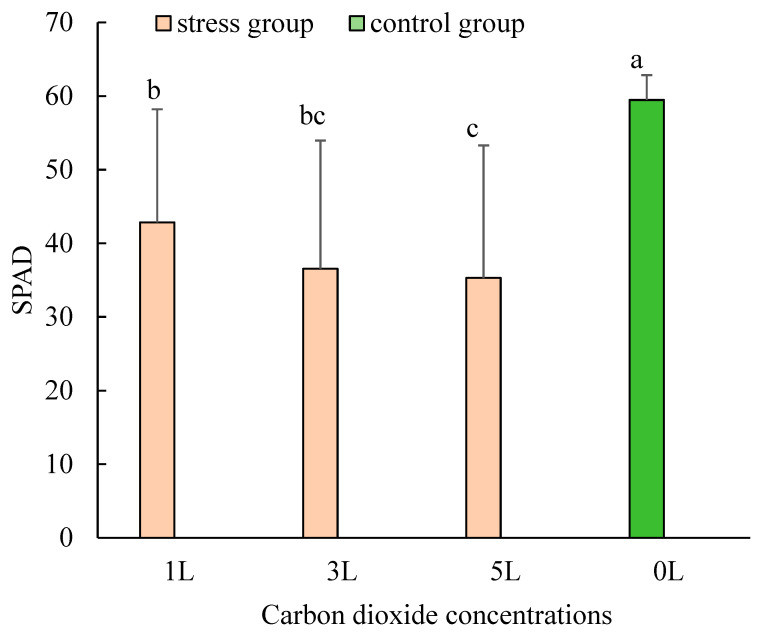
SPAD of wheat under different CO_2_ concentration gradients. Different lowercase letters indicate significant (*p* < 0.05) differences among the four stress rate levels under different treatments.

The maximum (max), minimum (min), mean, and standard deviation (SD) values of wheat SPAD at different CO_2_ concentrations were calculated (Table 1). The maximum, minimum, and mean values of wheat SPAD under ventilated CO_2_ were lower than those of the control, and the highest value of SPAD of wheat leaves in the control group was 65.5. Among the three types of CO_2_ concentration ventilation, the minimum value of wheat SPAD under 1 L/min CO_2_ concentration was 10.2, and the maximum value of wheat SPAD was 62.0. The smallest SD value was obtained in the case of ventilated wheat SPAD under 1 L/min; it can be seen in Figure 5 that the wheat leaves in the control group had the highest value of 65.5, while those in the control group had the lowest value of 51.1. The SPAD of wheat leaves in the control group was the highest, indicating the best growth of wheat in the natural ambient air. The 5 L/min treatment had the lowest SPAD, which was obvious from the results that, with the increase in the input CO_2_ concentration, there was a significant gradient decrease in the SPAD of wheat leaves, control > 1 L/min > 3 L/min > 5 L/min, indicating that with the increase in CO_2_ concentration, wheat leaves were increasingly affected by CO_2_, and wheat leaves were increasingly damaged, leading to a decrease in chlorophyll content.

### 3.2. Effects of Different Concentrations of CO_2_ on the Raw Hyperspectral Features of Wheat

**Figure 6:**
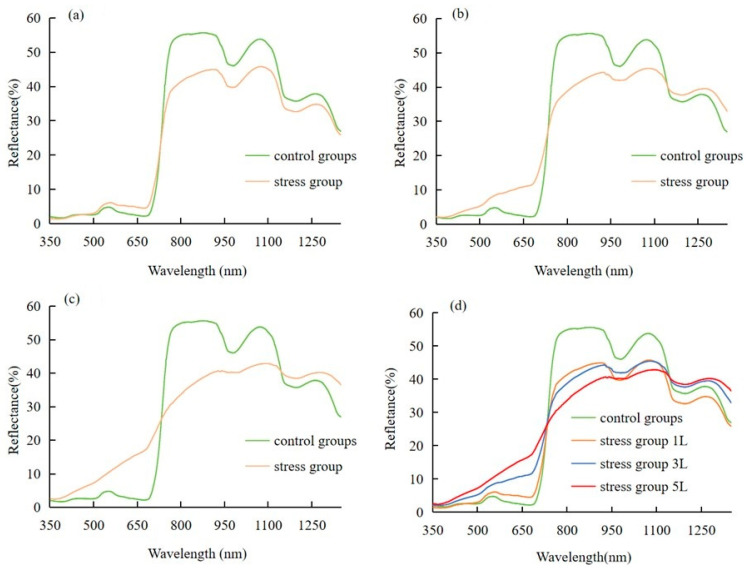
Spectral reflectance curves of wheat leaves. (**a**) 1 L, (**b**) 3 L, (**c**) 5 L, (**d**) Comparison of leaf spectra of winter wheat under different stress treatments.

As can be seen in Figure 6, the hyperspectral reflectance curves of wheat leaves treated with different CO_2_ concentrations have similar basic characteristics, as follows: the reflectance increases around 550 nm in the green band, forming a peak—‘green peak’. In the red band, the emissivity decreases around 680 nm, forming a valley—‘red valley’. The reflectance of wheat leaves increased sharply in the band 700–750 nm, and the position with the largest growth rate formed a hyperspectral feature—‘red edge’—which was maintained at a high level thereafter. The height of the green peak (around 550 nm) was ranked as follows: control < 1 L/min < 3 L/min < 5 L/min. It was found that the height of the green peak was related to the gradient of CO_2_ concentration, and it was easy to distinguish between the spectral reflectance curves of SPAD wheats at different CO_2_ concentrations. Compared with the CO_2_ concentration in the natural environment, the elevation of hyperspectral reflectance of wheat leaves under the 5 L/min treatment was significantly higher than that of other treatments, indicating that wheat under 5 L/min treatment was subjected to the greatest CO_2_ stress.

In our experiments, we observed significant changes in the spectral characteristics of the wheat canopy at different CO_2_ leakage rates. These changes may be attributed to the physiological adaptation mechanisms of wheat plants in response to different CO_2_ concentrations. Specifically, an increase in the concentration of CO_2_, which is the main raw material for photosynthesis, may promote the rate of photosynthesis and lead to changes in chlorophyll content and photosynthetic pigment distribution, which in turn affects the spectral reflectance characteristics [16]. These changes are particularly pronounced in the red and near-infrared regions, as chlorophyll absorption and reflection in these bands are important indicators of photosynthetic efficiency [36]. Furthermore, high CO_2_ concentrations may also indirectly alter the spectral characteristics of leaves by affecting physiological processes such as stomatal conductance and transpiration. The combined effect of these physiological mechanisms enables us to monitor and assess the growth status and stress response of wheat under different CO_2_ concentrations using spectral analysis.

### 3.3. SPAD-Sensitive Wavelength Selection Analysis

**Figure 7:**
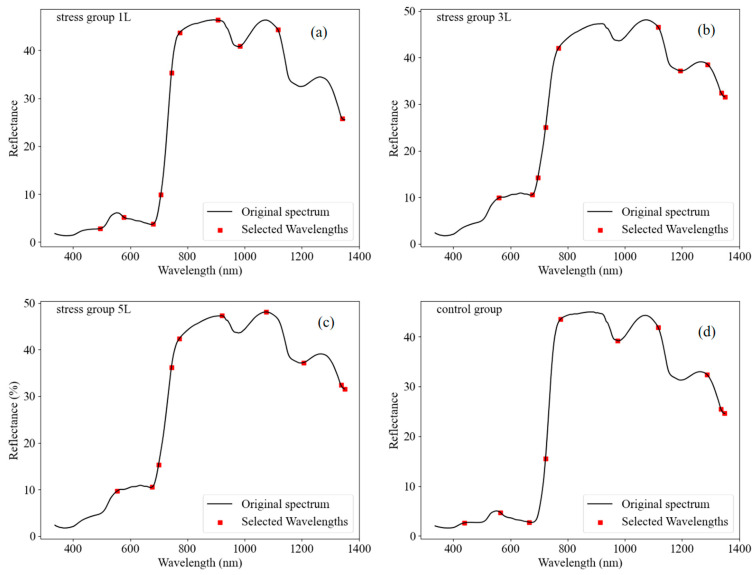
SPAD-sensitive wavelengths selected using SPA. (**a**–**d**) were the number of wavelengths selected by SPA method.

The results of SPA band selection are shown in Figure 7. We selected a total of 10 SPAD-sensitive bands as independent variables for the subsequent model. The distributions of the 10 bands are shown in Figure 7a–d. The x-axis denotes the wavelengths of 400–1350 nm, the y-axis denotes the reflectance of the original wavelengths, and the distributions of the ten bands are labelled by the red squares. It is worth noting that the first three bands marked with red squares correspond to the peaks and troughs of the reflectance curve. These bands were chosen based on their sensitivity to changes in chlorophyll content to capture the spectral signature of chlorophyll at specific wavelengths. Overall, these ten bands are evenly distributed across the spectrum.

**Figure 8:**
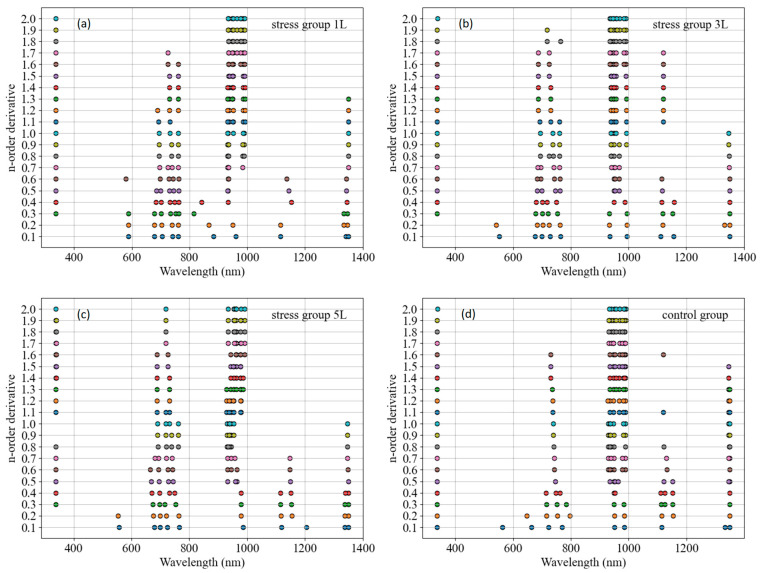
Wavelengths selected from fractional order differential spectra using the SPA method. (**a**) stress group 1 L, (**b**) stress group 3 L, (**c**) stress group 5 L, (**d**) control group were the selected wavelengths by SPA method from fractional order differential spectrum.

As a flexible mathematical tool, the fractional order derivative shows unique advantages in spectral analysis. Compared with the traditional integer order derivative, the fractional order derivative can describe the local features and trends in the spectral curves more finely [37]. In this study, by adjusting the order of the fractional order derivative, we were able to effectively extract spectral features highly correlated with the SPAD values of wheat, which may not be obvious or difficult to identify directly in the original spectra [38]. The introduction of the fractional order derivative not only improves the processing flexibility and information extraction ability of the spectral data, but also enhances the sensitivity of the model to subtle changes in the spectra, thus improving the accuracy and stability of the SPAD estimation model. In addition, the fractional order derivative can also suppress the interference of spectral noise to a certain extent, making the model more robust. The wavelengths selected from the fractional order differential spectra using the SPA method are shown in Figure 8. Under CO_2_ carbon stress, in the fractional order differential spectra, the wavelengths selected using the SPA method showed that the 0.1–0.2 order wavelengths around 400 nm were less selected, which may imply that the characteristics of these bands are less important for prediction under stress experimental conditions, and the rest of the wavelengths were in the near-infrared band. In Figure 8a–c, the wavelengths selected using the SPA method are distributed in the region after 600 nm, mainly in the regions of 600–800 nm and 900–1000 nm, and there are wavelengths within the first order of selection distributed in the region of 1100–1200 nm for all four treatment levels, which may be important for distinguishing the important information for predicting experimental results. These regions are usually associated with the water content of plant tissues, their cellular structure, and the indirect effects of pigments such as chlorophyll. Under CO_2_ stress conditions, changes in chlorophyll content may be reflected in spectral features in the near-infrared (NIR) band by affecting the water status and cellular structure of the plant [16]. In the control group, there were a small number of wavelengths between 700 and 800 nm at order 0.5–1.6, and all other wavelengths were after 900 nm (Figure 8d). Overall, the SPAD-sensitive wavelengths are rarely in the visible band, and most of them are in the NIR band except for the 0.1–0.4 order band; the SPAD-sensitive wavelengths are mainly after 900 nm, and most of the SPAD-sensitive wavelengths selected using the SPA method are in the NIR band as the fractional order increases. Increasing CO_2_ concentrations can affect the photosynthetic efficiency of plants, which in turn affects the rate of chlorophyll synthesis and degradation. At high CO_2_ concentrations, plants may increase their fixation of carbon, leading to changes in chlorophyll content or activity [16]. Such changes may be reflected by alterations in spectral characteristics, especially in the near-infrared (NIR) band, which is more sensitive to changes in the internal structure and water status of the plant [36]. The sensitive wavelengths selected in the NIR band using the SPA method may precisely capture these subtle spectral changes induced by CO_2_ stress, and thus serve as a key indicator for distinguishing the physiological status of plants under different stress conditions.

### 3.4. Modelling of SPAD Estimation in Wheat under CO_2_ Stress

**Figure 9:**
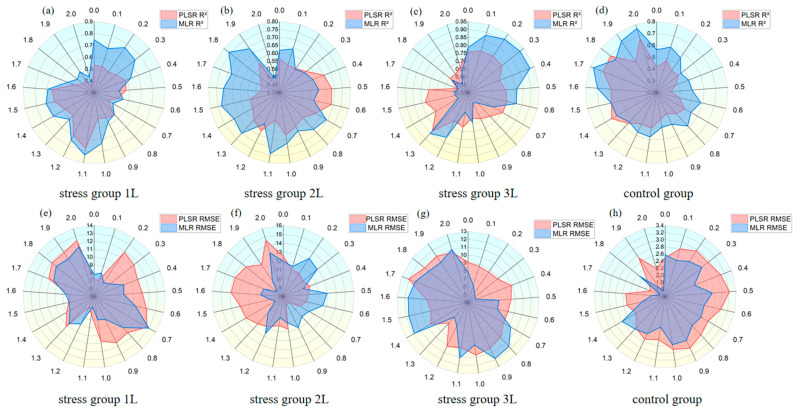
Comparison of test set inversion accuracy of fractional order differential SPAD models. (**a**–**d**) are R^2^ comparisons, (**e**–**h**) are RMSE comparisons.

The SPAD-sensitive wavelength was selected using the SPA method to establish the SPAD estimation model under CO_2_ stress. Based on the characteristic wavelengths selected from Figure 7 to Figure 8 as the independent variables of modelling parameters, the estimation model of SPAD content under CO_2_ stress was constructed using the measured values of SPAD in wheat leaves under different treatments as the dependent variable. The results are presented in Figure 9. The overall R^2^ of the test data set indicates that the PLSR model is inferior to the MLR model, while the RMSE indicates that the PLSR model is greater than the MLR model. For the 1 L/min, the PLSR model’s R^2^ is merely 0.058 higher than the MLR model at order 0.5, and the RMSE is lower than that of the MLR model at order 0, 0.1, 0.7, 1.2, and 1.6, and for other orders, the MLR model is better than the PLSR model. For the 3 L/min, the PLSR model surpassed the MLR model at steps 0.3, 0.4, 0.5, 0.6, and 1.2. For the 5 L/min, the MLR model performed better than the PLSR model at steps 0–0.6, and the PLSR model was mostly superior at subsequent steps. For the control group, the PLSR model outperformed the MLR model only at orders 1.2 and 1.8. The results demonstrate that the MLR model possesses high accuracy and stability, and the inverse values can fit the measurement results well.

**Figure 10:**
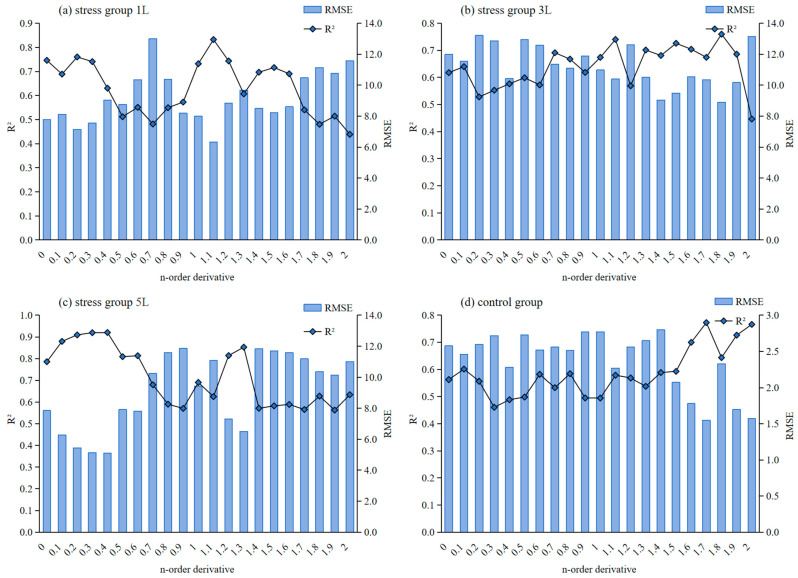
Wheat SPAD estimation model results. SPAD estimates for (**a**) stress group 1 L, (**b**) stress group 3 L, (**c**) stress group 5 L, (**d**) control group.

Figure 10 shows the fractional order difference in each fraction of R^2^ and RMSE for the SPAD-estimated MLR model under stress and control, where plots Figure 10a–d represent the metrics of the validation set accuracy test at different stress levels, respectively. The 1 L/min stress modelling results are shown in Figure 10a, from which it can be seen that the model accuracies after the 1.7 order all decreased; from the validation set of the accuracy indexes, it can be seen that the highest validation accuracy is the 1.1-order model, with an R^2^ of 0.832, which is 11.528% higher than that of the 0-order, and the RMSE is the lowest in the validation set, with an R^2^ of 6.321, which is 18.732% lower than that of the 0-order. The 3 L/min model results are shown in Figure 10b, which are similar to the 1 L/min model results, with a decrease in the 2.0-order accuracy; in the validation set, the 1.8-order model results are superior, with an R^2^ of 0.760, which is 14.2% higher than the 0-order, and an RMSE of 2.211, which is 25.9% lower than the 0-order.

The 5 L/min model results are shown in Figure 10c. The overall accuracy of the validation set is better, and the validation set is relatively better in order 0.1 to 0.4; in the validation set, the model is optimal in order 0.4, with an R^2^ of 0.92, which improves by 17.048% compared with the 0th order, and an RMSE of 5.106, which reduces by 34.997% compared with the 0th order. The results of the control group are shown in Figure 10d, where the accuracy of each fractional order model is improved compared to the 0th-order model. In the validation set, the 1.7-order is the optimal model with an R^2^ of 0.773, which is improved by 37.3% compared to the 0th-order.

**Figure 11:**
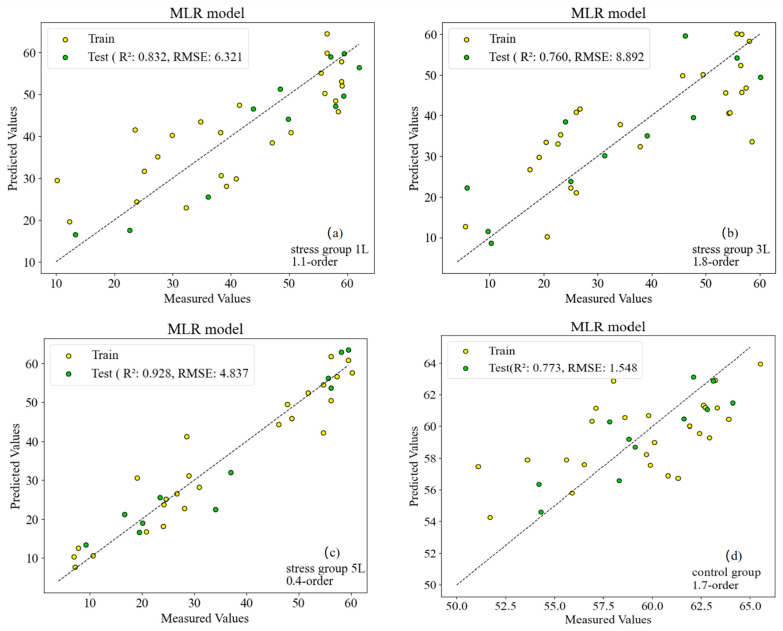
Comparison of (**a**) stress group 1 L, (**b**) stress group 3 L, (**c**) stress group 5 L, (**d**) control group measured and estimated SPAD values in stressed wheat.

Based on the comprehensive analysis results of the validation set results under each stress concentration, the optimal fractional order differential model under stress was selected, in which 1 L/min was the optimal estimation model with 1.1 order, 3 L/min was the optimal model with 1.8 order, 5 L/min was the optimal model with 0.4 order, and the control group was the optimal model with 1.7 order. Based on the results of each stress, the optimal fractional order differential estimation was used. The results of the model validation set were plotted as scatter plots of measured and predicted SPAD values of wheat leaves, with the solid line representing the fitted line and the dashed line representing the 1:1 line, and the results are shown in Figure 11.

As shown in Figure 11, the fitted lines of the scatter plots were observed to be closer to the dotted line under each concentration of CO_2_ stress, indicating a certain degree of accuracy in the estimation models. The scatter plots for 1 L/min and 5 L/min were relatively evenly distributed on both sides of the dotted line, while most of the scatter plots for 3 L/min and the control group were situated above the dotted line. This suggests that the predicted values of the estimation models for 1 L/min and the control group were relatively larger compared to the measured values, resulting in a relatively poor validation accuracy of them in the four models. Upon comparison between 1 L/min and 5 L/min, it is evident that their validation accuracies are comparable with R^2^ values above 0.75, placing them in a higher grade among all four models.

Fractional differentiation can disclose diverse levels of detailed information in spectral data. Lower fractional orders (e.g., 0.1–0.4) might preserve more of the smoothing characteristics of the original spectrum while highlighting minute alterations in the spectrum, which could be more susceptible to certain specific stress responses. Nevertheless, higher fractional orders (such as those exceeding 1.7) might introduce excessive noise or overly parse spectral information, leading to a reduction in model accuracy.

As depicted in Figure 11, the fractional order of the optimal model varies under different stress levels. This implies that the spectral characteristics of plant leaves under different stress conditions are specific, and it is necessary to select an appropriate fractional order to capture these variations. Hence, the selection of the fractional order should be adjusted in accordance with the specific stress conditions during model construction. Firstly, in this study, we employed SPA for feature selection to extract the bands that are most sensitive to chlorophyll content from hyperspectral data. The distinct band combinations selected using SPA (e.g., 1.1, 1.8, 0.4, and 1.7 differential) exhibit dissimilar model performances at different CO_2_ leakage rates. This indicates that the feature selection method can effectively reduce data redundancy and enhance the prediction accuracy of the model. However, the performance disparities among the different feature combinations might reflect subtle changes in the spectral properties of wheat leaves at different CO_2_ leakage rates, which might affect the sensitivity and accuracy of the model regarding chlorophyll content. Secondly, we constructed both MLR and PLSR models and discovered that the MLR model performed better in most cases. This might be because the MLR model is more efficient when dealing with data sets featuring linear relationships, and our data sets might be more linear after fractional differentiation processing. In contrast, PLSR, although capable of handling the relationship between multiple independent and dependent variables concurrently, might not be as flexible or accurate as MLR models in certain circumstances. Therefore, the choice of model type and the matching of data characteristics are crucial factors influencing the performance of the model. As the CO_2_ leakage rate increases (particularly from 1 L/min to 5 L/min), wheat leaves are more significantly influenced and model performance improves (e.g., the validation set R² value increases). This could be attributed to more pronounced changes in the spectral properties of wheat leaves at higher CO_2_ leakage rates, and these changes provide the model with more information to accurately predict chlorophyll content. However, it might also signify that the generalization ability of the model requires further validation under extreme environmental conditions.

## 4. Discussion

The SPAD of plant leaves reflects the growth level of the plant, determines its productivity, and is a good indicator of plant growth status [39]. Different levels of CO_2_ concentration have different effects on wheat leaves and reflect different physiological and ecological characteristics. In this study, wheat grown at 1 L/min was already persecuted by CO_2_, and as the CO_2_ concentration increased, the SPAD of wheat leaves was subsequently reduced. Until the CO_2_ concentration was increased to 5 L/min, the intensity of persecution of wheat by high CO_2_ concentration increased. Previous studies have shown that elevated CO_2_ concentration has different effects on the chlorophyll content of wheat at different CO_2_ concentrations. Zhou Ning et al. [40] used an on-farm T-FACE system to monitor the chlorophyll content of rice grown in a 550 ${\mu moL\cdot mol}^{-1}$ environment, and found that in the early stage of the rice season, the chlorophyll content significantly increased, and then in the later stage, the chlorophyll content reduced rapidly. It was found that the chlorophyll content was significantly increased in the early stage of rice, and then rapidly decreased in the later stage, resulting in the phenomenon of ‘early senescence’. Using the OTC system, Wang Peiling et al. [41] found that the chlorophyll content of winter wheat leaves decreased after doubling the CO_2_ concentration, but the decrease varied according to the fertility stage. The increase in soil CO_2_ concentration inhibits plant growth more than any atmospheric greenhouse effect [42,43]. Previous studies have shown that high soil CO_2_ concentrations inhibit photosynthesis in plants such as forage, alfalfa, and soybeans, resulting in shorter plant heights, thinner stems, lower biomass, and lower chlorophyll content [44,45]. From Figure 3, it can be clearly seen that the middle wheat obviously turned yellow under 5 L/min leakage rate, but the maximum concentration and time that plants can tolerate CO_2_ persecution are not yet conclusive, and further research can be conducted in this area in the future.

Compared to the near-infrared band interval, chlorophyll absorbs visible light in the short-wave interval and mainly absorbs blue and red light, so it will form a red valley, and because chlorophyll does not absorb as much green light as other colours, the leaf is green and the spectral curve shows green peaks [46]. The hyperspectral features of wheat leaves changed under CO_2_ stress, and with the increase in the concentration of CO_2_, the hyperspectral features of wheat leaves changed more obviously, as shown in Figure 6. From the original spectral curves, it can be learnt that the higher the CO_2_ concentration, the higher the green peak. The green peak corresponds to the reflectance of the green light band reflected from the inside of the wheat leaf. The higher the reflectance, the less chlorophyll content inside the wheat leaf, the lower the absorption rate, and the less healthy the wheat leaf is [47]. The more CO_2_-infested wheat leaves, the more reflections in the green band and the higher green peaks than under other treatments. This indicates that the stress on wheat increased with the increase in CO_2_ concentration, which is consistent with previous findings. Keith et al. [48] showed that the reflectance of alfalfa changed significantly at 650–750 nm under soil CO_2_ stress. Chen [49] et al. demonstrated that the reflectance of sugar beet leaves under CO_2_ leakage stress was decreased at 550 nm and increased at 680 nm.

For the final SPAD estimation model of stressed wheat, the results show that the fractional order differential transform improved the accuracy of the SPAD estimation model (Figure 11) with better results. The implementation of the method was simple and efficient, and an accuracy of more than 0.90 for SPAD estimation in stressed wheat could be obtained using only ten wavelengths (Figure 8c). Comparing the independent variable composition and stability of the MLR estimation model at four CO_2_ concentration levels, elevated CO_2_ concentration did not affect the construction of the SPAD estimation model for winter wheat leaves. By comparing the estimation models at different CO_2_ leakage rates, we found that all of them had good estimation accuracy, but the accuracy and stability of the 5 L/min model were better than those of the other leakage rates and the control model, and the R^2^ of the estimation model could reach 0.928. Although the estimation model of SPAD in winter wheat in the present study achieved good results, the applicability of the model remains to be demonstrated in the future due to the number of years of the experiments and the relatively small number of samples. However, due to the small number of years and samples in the experiment, the applicability of the model needs to be verified. In conclusion, the present study shows the feasibility of the MLR model for estimating the SPAD of winter wheat under CO_2_ microleakage.

In practice, the specific impact of CO_2_ microleakage on wheat growth can be assessed by comparing the changes in the spectral curves of wheat leaves (e.g., the ‘redshift’ phenomenon of the green peak and the ‘blueshift’ phenomenon of the red margins) at different CO_2_ leakage rates. This helps farmers and agricultural managers to identify environmental stresses in time and take appropriate management measures. Based on the chlorophyll content results predicted using the MLR model, farmers can develop more precise irrigation and fertilization strategies to meet crop growth needs at different CO_2_ leakage rates. For example, at higher CO_2_ leakage rates, the amount of nitrogen fertilizer applied can be increased appropriately to mitigate the adverse effects of stress on crop growth. By monitoring the growth performance of different wheat varieties under CO_2_ microleakage conditions over a long period of time, combined with data on changes in chlorophyll content, it is possible to screen out crop varieties that are more resilient and better able to adapt to future climate change. This could help to improve the stability and sustainability of agricultural production.

## 5. Conclusions

In this study, wheat canopy hyperspectral reflectance data were effectively processed and analyzed by combining the fractional order differentiation method and SPA feature selection technique, aiming to improve the estimation accuracy of SPAD values in CO_2_-stressed wheat. The main conclusions are as follows:

(1) Successful pre-processing of hyperspectral data using fractional order differentiation significantly improved the ability of the MLR model to estimate the SPAD values of wheat under different CO_2_ leakage rates (1 L/min, 3 L/min, 5 L/min, 0 L/min). The best models were based on 1.1, 1.8, 0.4 and 1.7 orders of differentiation, which improved the R² values over the original spectral model on the validation set by 11.528%, 14.2%, 17.048%, and 37.3%, respectively, indicating that appropriate spectral transformations can effectively improve the model’s performance.
(2) The SPA method was used to accurately screen the feature wavelengths that were highly sensitive to wheat SPAD from the huge amount of spectral information, and these feature wavelengths played a key role in MLR modelling, further demonstrating the importance of feature selection for improving model efficiency and accuracy.

In this study, the feature selection technique was integrated into hyperspectral technology for the first time to achieve the accurate estimation of wheat’s SPAD value under a CO_2_ microleakage environment, which provides a solid theoretical foundation and technical support for monitoring vegetation stress information and locating CO_2_ leakage points using hyperspectral remote sensing. More advanced spectral processing methods and feature selection algorithms will be explored in the future to further improve the model’s accuracy and generalization ability. We will expand the experimental scope to study the spectral response characteristics under different crop species, growing conditions, and CO_2_ leakage concentration. We will combine these with remote sensing platforms, such as drones and satellites, to achieve large-scale high temporal and spatial resolution monitoring of vegetation stress and CO_2_ leakage.

## Figures and Tables

**Table 1:** Wheat leaf SPAD statistics.

Indicators	Stress Group 1 L	Stress Group 3 L	Stress Group 5 L	Control Group
min	10.2	5.5	7.0	51.1
max	62.0	60.1	60.2	65.5
mean	42.9	36.6	35.3	59.5
SD	15.6	17.7	18.3	3.7

## Data Availability

The data presented in this study are available on request from the corresponding author. The data are not publicly available due to privacy.

## References

[1] Liu Q., Wu S., Lei Y., Li S., Li L. (2021). Exploring Spatial Characteristics of City-Level CO_2_ Emissions in China and Their Influencing Factors from Global and Local Perspectives. Sci. Total Environ..

[2] Luo F., Guo Y., Yao M., Cai W., Wang M., Wei W. (2020). Carbon Emissions and Driving Forces of China’s Power Sector: Input-Output Model Based on the Disaggregated Power Sector. J. Clean. Prod..

[3] Duan C., Zhu W., Wang S., Chen B. (2022). Drivers of Global Carbon Emissions 1990–2014. J. Clean. Prod..

[4] Metz B., Davidson O., de Coninck H., Loos M., Meyer L. (2005). IPCC Special Report on Carbon Dioxide Capture and Storage.

[5] Pearce J., Jones D., Blackford J., Beaubien S., Foekema E., Gemeni V., Kirk K., Lions J., Metcalfe R., Moni C. (2014). A Guide for Assessing the Potential Impacts on Ecosystems of Leakage from CO_2_ Storage Sites. Energy Procedia.

[6] Zhou X., Apple M.E., Dobeck L.M., Cunningham A.B., Spangler L.H. (2013). Observed Response of Soil CO_2_ Concentration to Leaked CO_2_ from an Engineered CO_2_ Leakage Experiment. Int. J. Greenh. Gas Control.

[7] Chen X., Li F., Shi B., Fan K., Li Z., Chang Q. (2023). Estimation of Winter Wheat Canopy Chlorophyll Content Based on Canopy Spectral Transformation and Machine Learning Method. Agronomy.

[8] Wang T., Gao M., Cao C., You J., Zhang X., Shen L. (2022). Winter Wheat Chlorophyll Content Retrieval Based on Machine Learning Using in Situ Hyperspectral Data. Comput. Electron. Agric..

[9] Cai Y., Miao Y., Wu H., Wang D. (2021). Hyperspectral Estimation Models of Winter Wheat Chlorophyll Content Under Elevated CO_2_. Front. Plant Sci..

[10] Gao G., Zhang L., Wu L., Yuan D. (2024). Estimation of Chlorophyll Content in Wheat Based on Optimal Spectral Index. Appl. Sci..

[11] Chen Z., Hao P., Liu J., An M., Han B. (2019). Technical demands for agricultural remote sensing satellites in China. Smart Agric..

[12] Lan Y., Wang T., Chen S. (2020). Agricultural artificial intelligence technology: Wings of modern agricultural science and technology. J. South China Agric. Univ..

[13] Xu D., Liu X., Wang W., Chen M., Kan H. (2017). Hyper-spectral characteristics and estimation model of leaf chlorophyll content in cotton under waterlogging stress. Chin. J. Appl. Ecol..

[14] Xia T., Yang K., Feng F., Guo H., Zhang C. (2021). A New Copper Stress Vegetation Index NCSVI Explores the Sensitive range of Corn Leaves Spectral under Copper Pollution. Spectrosc. Spectr. Anal..

[15] Goswami J., Das R., Sarma K.K., Raju P. (2021). Red Edge Position (REP), an Indicator for Crop Stress Detection: Implication on Rice (*Oryza sativa* L). Int. J. Environ. Clim. Change.

[16] Noomen M.F., Skidmore A.K. (2009). The Effects of High Soil CO_2_ Concentrations on Leaf Reflectance of Maize Plants. Int. J. Remote Sens..

[17] Lakkaraju V.R., Zhou X., Apple M.E., Cunningham A., Dobeck L.M., Gullickson K., Spangler L.H. (2010). Studying the Vegetation Response to Simulated Leakage of Sequestered CO_2_ Using Spectral Vegetation Indices. Ecol. Inform..

[18] Tuerxun N., Zheng J., Wang R., Wang L., Liu L. (2023). Hyperspectral Estimation of Chlorophyll Content in Jujube Leaves: Integration of Derivative Processing Techniques and Dimensionality Reduction Algorithms. Front. Plant Sci..

[19] Zhang Y., Chang Q., Chen Y., Liu Y., Jiang D., Zhang Z. (2023). Hyperspectral Estimation of Chlorophyll Content in Apple Tree Leaf Based on Feature Band Selection and the CatBoost Model. Agronomy.

[20] Broge N.H., Leblanc E. (2001). Comparing Prediction Power and Stability of Broadband and Hyperspectral Vegetation Indices for Estimation of Green Leaf Area Index and Canopy Chlorophyll Density. Remote Sens. Environ..

[21] Li P., Huang X., Yin S., Bao Y., Bao G., Tong S., Dashzeveg G., Nanzad T., Dorjsuren A., Enkhnasan D. (2023). Optimizing Spectral Index to Estimate the Relative Chlorophyll Content of the Forest under the Damage of Erannis Jacobsoni Djak in Mongolia. Ecol. Indic..

[22] Zhang J., Tian H., Wang D., Li H., Mouazen A.M. (2021). A Novel Spectral Index for Estimation of Relative Chlorophyll Content of Sugar Beet. Comput. Electron. Agric..

[23] Li C., Wang Y., Ma C., Ding F., Li Y., Chen W., Li J., Xiao Z. (2021). Hyperspectral Estimation of Winter Wheat Leaf Area Index Based on Continuous Wavelet Transform and Fractional Order Differentiation. Sensors.

[24] Zhao Q., Ge X., Ding J., Wang J., Zhang Z., Tian M. (2020). Combination of Fractional Order Differential and Machine Learning Algorithm for Spectral Estimation of Soil Organic Carbon Content. Ji Guang Yu Guang Dian Zi Xue Jin Zhan.

[25] Li C., Ma C., Chen P., Cui Y., Shi J., Wang Y. (2021). Machine Learning-Based Estimation of Potato Chlorophyll Content at Different Growth Stage Using UAV Hyperspectral Data. Zemdirb.-Agric..

[26] Liu Y., Feng H., Sun Q., Yang F., Yang G. (2020). Estimation of Potato Above-ground Biomass Based on Fractional Differential of UAV Hyperspectral. Trans. Chin. Soc. Agric. Mach..

[27] Wang S., Chen Y., Wang M., Zhao Y., Li J. (2019). SPA-Based Methods for the Quantitative Estimation of the Soil Salt Content in Saline-Alkali Land from Field Spectroscopy Data: A Case Study from the Yellow River Irrigation Regions. Remote Sens..

[28] Saberioon M.M., Amin M.S.M., Anuar A.R., Gholizadeh A., Wayayok A., Khairunniza-Bejo S. (2014). Assessment of Rice Leaf Chlorophyll Content Using Visible Bands at Different Growth Stages at Both the Leaf and Canopy Scale. Int. J. Appl. Earth Obs. Geoinf..

[29] Pan Y., Jiang J., Li K., Xiong K., Du Y., Wang X. (2023). Identificating Vegetation Stress under Natural Gas Micro-Leakage Based on Leaf Scale Temporal Hyperspectrum. Int. J. Remote Sens..

[30] Wu R., Fan Y., Zhang L., Yuan D., Gao G. (2024). Wheat Yield Estimation Study Using Hyperspectral Vegetation Indices. Appl. Sci..

[31] Zhang J., Jing X., Song X., Zhang T., Duan W., Su J. (2023). Hyperspectral Estimation of Wheat Stripe Rust Using Fractional Order Differential Equations and Gaussian Process Methods. Comput. Electron. Agric..

[32] Liu Y., Zhang Y., Lu H., Yang Y., Xie J., Chen D. (2024). Application of Fractional-Order Differential and Ensemble Learning to Predict Soil Organic Matter from Hyperspectra. J. Soils Sediments.

[33] Hong Y., Chen Y., Yu L., Liu Y., Liu Y., Zhang Y., Liu Y., Cheng H. (2018). Combining Fractional Order Derivative and Spectral Variable Selection for Organic Matter Estimation of Homogeneous Soil Samples by VIS–NIR Spectroscopy. Remote Sens..

[34] Zhang D., Guo Q., Cao L., Zhou G., Zhang G., Zhan J. (2021). A Multiband Model with Successive Projections Algorithm for Bathymetry Estimation Based on Remotely Sensed Hyperspectral Data in Qinghai Lake. IEEE J. Sel. Top. Appl. Earth Obs. Remote Sens..

[35] Zelterman D., Zelterman D. (2022). Multivariable Linear Regression. Applied Multivariate Statistics with R.

[36] Ko D., Yoo G., Yun S.-T., Chung H. (2016). Impacts of CO_2_ Leakage on Plants and Microorganisms: A Review of Results from CO_2_ Release Experiments and Storage Sites. Greenh. Gases Sci. Technol..

[37] Dou S., Zhang W., Deng Y., Zhang C., Mei Z., Yan J., Li M. (2024). Comparison of Citrus Leaf Water Content Estimations Based on the Continuous Wavelet Transform and Fractional Derivative Methods. Horticulturae.

[38] Xiao B., Li S., Dou S., He H., Fu B., Zhang T., Sun W., Yang Y., Xiong Y., Shi J. (2024). Comparison of Leaf Chlorophyll Content Retrieval Performance of Citrus Using FOD and CWT Methods with Field-Based Full-Spectrum Hyperspectral Reflectance Data. Comput. Electron. Agric..

[39] Shi T., Liu H., Chen Y., Wang J., Wu G. (2016). Estimation of Arsenic in Agricultural Soils Using Hyperspectral Vegetation Indices of Rice. J. Hazard. Mater..

[40] Zhou N., Jing L., Wang Y., Zhu J., Yang L., Wang Y. (2017). Effects of Elevated Atmospheric CO_2_ and Temperature on Dynamics of Leaf Chlorophyll Contents and SPAD Value of Rice in Open-Air Field Conditions. Chin. J. Rice Sci..

[41] Wang P., Xu Y., Song S., Shen Y., Li S. (2011). Effect of Doubled Atmospheric CO_2_ and Nitrogen Application on Photosynthetic Rate and Chlorophyll Fluorescence Character of Winter Wheat. Acta Bot. Boreali-Occident. Sin..

[42] Stenhouse M., Arthur R., Zhou W. (2009). Assessing Environmental Impacts from Geological CO_2_ Storage. Energy Procedia.

[43] Paulley A., Metcalfe R., Egan M., Maul P.R., Limer L., Grimstad A.-A. (2013). Hypothetical Impact Scenarios for CO_2_ Leakage from Storage Sites. Energy Procedia.

[44] Wu Y., Ma X., Li Y.E., Wan Y.F. (2014). The Impacts of Introduced CO_2_ Flux on Maize/Alfalfa and Soil. Int. J. Greenh. Gas Control.

[45] Patil R.H., Colls J.J., Steven M.D. (2010). Effects of CO_2_ Gas as Leaks from Geological Storage Sites on Agro-Ecosystems. Energy.

[46] Virtanen O., Constantinidou E., Tyystjärvi E. (2020). Chlorophyll Does Not Reflect Green Light—How to Correct a Misconception. J. Biol. Educ..

[47] Xie J., Zhou S., Chung L.C.H., Chan T.O. (2024). Evaluating Land-Surface Warming and Cooling Environments across Urban–Rural Local Climate Zone Gradients in Subtropical Megacities. Build. Environ..

[48] Keith C.J., Repasky K.S., Lawrence R.L., Jay S.C., Carlsten J.L. (2009). Monitoring Effects of a Controlled Subsurface Carbon Dioxide Release on Vegetation Using a Hyperspectral Imager. Int. J. Greenh. Gas Control.

[49] Chen Y.H., Jiang J.B., A-Du G., Li Y.F. (2012). Research on the Spectral Feature and Identification of the Surface Vegetation Stressed by Stored CO_2_ Underground Leakage. Guang Pu Xue Yu Guang Pu Fen Xi = Guang Pu.

